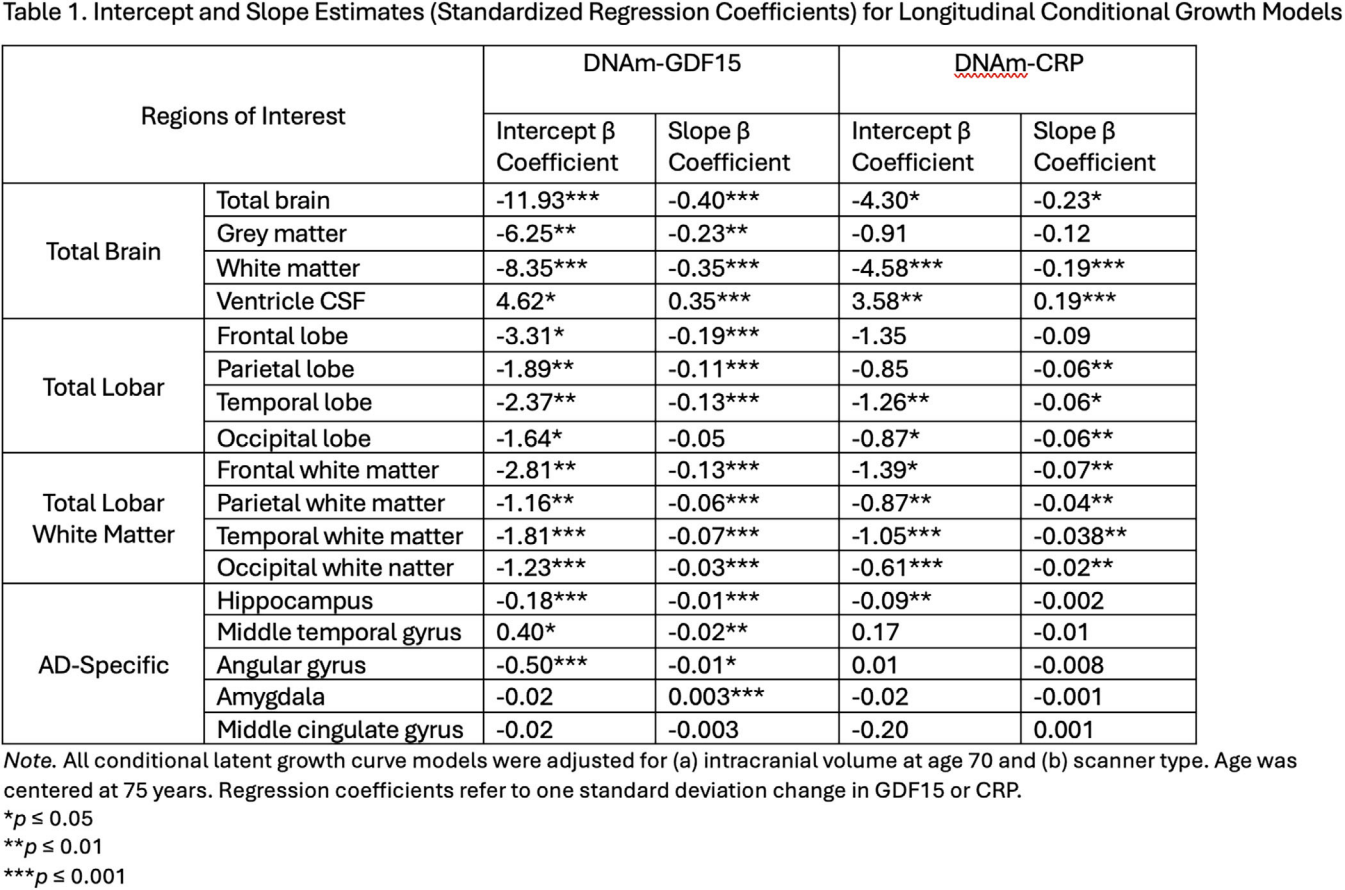# CRP and GDF15 DNA methylation signatures differentially predict brain volume loss

**DOI:** 10.1002/alz.095367

**Published:** 2025-01-09

**Authors:** Shannon M. Drouin, Pei‐Lun Kuo, Zenobia Moore, Christos Davatzikos, Susan M. Resnick, Keenan A. Walker

**Affiliations:** ^1^ Laboratory of Behavioral Neuroscience, National Institute on Aging, Intramural Research Program, Baltimore, MD USA; ^2^ Translational Gerontology Branch, National Institute on Aging, Intramural Research Program, Baltimore, MD USA; ^3^ Artificial Intelligence in Biomedical Imaging Laboratory (AIBIL), Center for and Data Science for Integrated Diagnostics (AI2D), Perelman School of Medicine, University of Pennsylvania, Philadelphia, PA USA

## Abstract

**Background:**

C‐reactive protein (CRP) and growth differentiation factor 15 (GDF15) are important markers of inflammation associated with several aging‐related morbidities. DNA methylation (DNAm) measures of CRP and GDF15 may provide stable epigenetic measures of chronic exposure to inflammation and could therefore be robustly predictive of inflammation‐related brain aging and neurodegeneration.

**Methods:**

Data from a subsample of cognitively normal older adults (*n* = 431) from the Baltimore Longitudinal Study of Aging with DNA methylation (DNAm) data and longitudinal structural neuroimaging (up to 8 visits, 16.4 years) were used. We used latent growth curve models (Mplus 8.2) to explore the effect of two DNAm signatures (DNAm‐GDF15 and DNAm‐CRP) on longitudinal trajectories of 17 brain region volumes. These included total brain, total lobar, total lobar white matter, and other regions vulnerable to AD‐pathology.

**Results:**

Our analyses revealed several brain regions in which level (at age 75) and change were significantly predicted by DNAm‐GDF15 and DNAm‐CRP (Table 1). Specifically, higher DNAm‐GDF15 predicted lower volumes and greater decline in the total brain (β_i_ = ‐11.93; β_s_ = ‐0.40), total grey matter (β_i_ = ‐6.25; β_s_ = ‐0.23), and total white matter regions (β_i_ = ‐8.35; β_s_ = ‐0.35), as well as greater ventricular volume and increases (β_i_ = 4.62; β_s_ = 0.35). Our analyses also revealed broad effects of GDF15 on total lobar, total lobar white matter, and AD‐specific regions. Our second set of conditional growth models revealed that higher DNAm‐CRP predicted lower volumes and greater decline in the total brain (β_i_ = ‐4.30; β_s_ = ‐0.23) and total white matter regions (β_i_ = ‐4.58; β_s_ = ‐0.19), as well as greater ventricular volume and increases (β_i_ = 3.58; β_s_ = 0.19). DNAm‐CRP also broadly predicted level and decline in volume for several total lobar and total lobar white matter regions, but these effects did not extend to AD‐specific regions.

**Conclusions:**

In a sample of cognitively normal older adults, epigenetic signatures representing longer‐term exposure to two inflammatory proteins differentially predicted brain volume trajectories. Notably, DNAm measures of GDF15 showed particularly strong associations with a broader breadth of brain regions as compared to DNAm‐CRP.